# Ultra-High-Density Catheter Ablation of Purkinje Premature Ventricular Contractions Causing Electrical Storm

**DOI:** 10.1016/j.jaccas.2024.102789

**Published:** 2025-01-15

**Authors:** Pablo Vadillo Martín, Mercedes Cabrera Ramos, Isabel Montilla Padilla, Jorge Melero Polo, Javier Ramos Maqueda

**Affiliations:** Hospital Clínico Universitario Lozano Blesa, Zaragoza, Spain

**Keywords:** ablation mapping catheter, polymorphic ventricular tachycardia, premature ventricular contractions, Purkinje

## Abstract

The postacute myocardial infarction electrical storm is a life-threatening entity. Resistance to ischemia in Purkinje fibers may be the origin of short-coupled premature ventricular contractions that trigger severe arrhythmic events. We present a case where the use of emergency catheter ablation, guided by a 3D navigation system and an ultra-high-density mapping catheter, successfully terminated the arrhythmic storm. This case highlights the crucial role of advanced mapping and ablation techniques in managing complex postinfarction arrhythmias and provides valuable insights that may enhance future clinical practice in similar critical situations.

## Graphical Abstract

**Figure:**
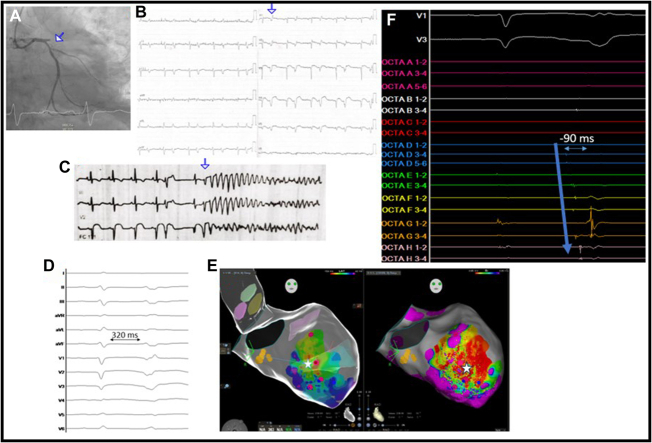

We present the case of a 60-year-old man with diabetes mellitus as cardiovascular risk factor, who attended the emergency room because of asthenia and poor general condition after suffering an episode of chest pain 10 days earlier. The electrocardiogram showed ST-segment elevation in anterior leads with established Q-wave, leading to the diagnosis of evolved acute anterior ST-segment elevation myocardial infarction. Given the presence of severe heart failure, emergent coronary angiography was performed, revealing subacute thrombotic occlusion (TIMI 0) at the proximal level of the anterior descending artery (Figure 1A). Percutaneous coronary intervention was performed with manual thrombus aspiration, but the distal flow was not restored.

**Figure 1:**
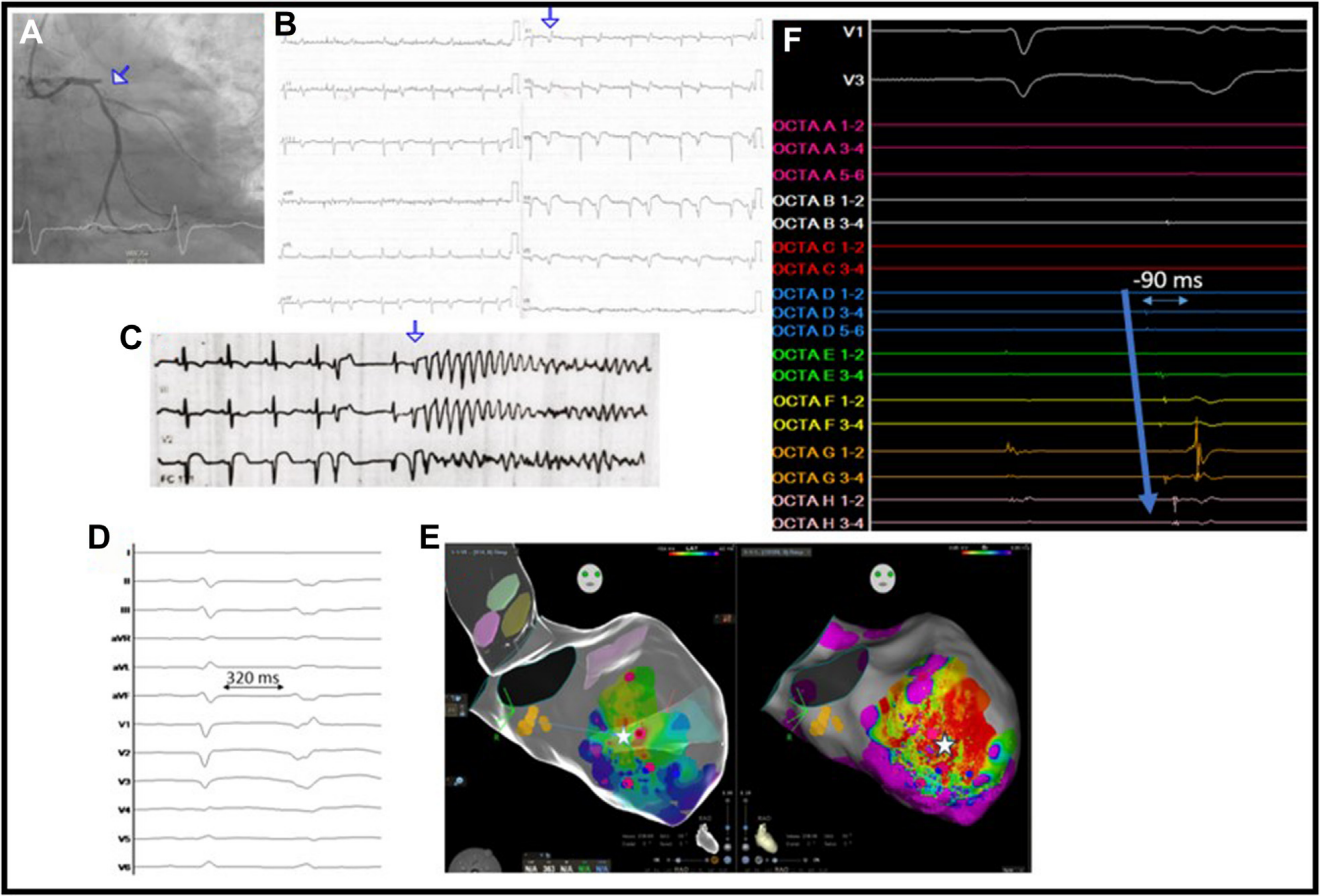
Ablation of Purkinje Premature Ventricular Contractions (A) Emergency coronary angiography reveals acute thrombotic occlusion at the proximal level of the anterior descending artery. (B) 12-Lead electrocardiogram displays sinus rhythm, anterior Q-wave with the persistence of anterior elevation of the ST segment, and bigeminy short-coupled premature ventricular contractions (PVCs) originating in the posterior fascicle. (C) Polymorphic ventricular tachycardia triggered by short-coupled PVC. (D) PVC originating in the posterior fascicle and with a coupling interval of 320 ms. (E) Endocardial bipolar voltage map shows scar tissue in the septal area with areas of low voltage affecting the conduction system and the Purkinje network of the left bundle branch. (F) Activation map, from distal to proximal, demonstrates the Purkinje potential preceding the ventricular PVC with −90 ms precocity.

On the sixth day of admission, the patient developed an electrical storm, characterized by repeated episodes of polymorphic ventricular tachycardia (PVT) and multiple defibrillations, despite antiarrhythmic treatment (amiodarone, quinidine, beta-blockers) and deep sedation with intubation (Figures 1B and 1C). All episodes were triggered by monomorphic short-coupled premature ventricular contractions (PVCs) with a coupling interval of 320 ms, with electrocardiographic morphology suggesting an origin in the posterior fascicle (Figure 1D). Therefore, endocardial ablation was considered to map and suppress the PVCs.

High-resolution mapping with 10,700 points of the left ventricle was conducted using an ultra-high-density mapping catheter (OctaRay, Biosense Webster) and a 3D navigation system (Carto 3, Biosense Webster). The endocardial bipolar voltage map showed scar tissue in the septal area, with low-voltage areas affecting the conduction system and the Purkinje network of the left bundle branch (Figure 1E). An activation map was obtained, revealing Purkinje potentials in the area of earliest activation, preceding the target PVC by 90 ms (Figure 1F).

The ablation was performed with a contact-force catheter (Smart Touch, BioSense Webster), applying an ablation index (AI) of 500 at 35 W at the identified site. The ablation index (AI) is a combined value that takes into account the power, application time, and contact force of the catheter on the tissue. In this case, an AI of 500 indicates a specific energy level considered adequate to achieve an effective lesion without compromising the safety of the procedure. There were no episodes of ventricular fibrillation during the procedure, but there was high-density ectopy, which was completely abolished after the radiofrequency applications, and we reinforced the area to achieve complete scar homogenization. There were no immediate complications. After the procedure, antiarrhythmic agents were discontinued and the patient had a favorable outcome during hospitalization with no more arrhythmic events. However, because of advanced heart failure despite optimal medical management, a long-term ventricular assist device was implanted. At the time of writing, the patient was in NYHA functional class I with no further hospitalizations.

The postacute myocardial infarction electrical storm is a life-threatening entity. The resistance to ischemia of the Purkinje fibers may be the origin of short-coupled PVCs that trigger severe arrhythmic events, including PVT and ventricular fibrillation, owing to automatism secondary to increased activity triggered by these fibers.^1^, ^2^, ^3^ The response to conventional pharmacologic treatment is often inadequate, and emergency ablation procedures should be considered. This case illustrates the effectiveness of emergency catheter ablation, using a 3D navigation system combined with an ultra-high-density mapping catheter, in safely and effectively managing the arrhythmia. The successful resolution of this critical scenario underscores the importance of advanced interventional techniques in the treatment of complex postinfarction arrhythmias, offering valuable guidance for future clinical practice.

## Funding Support and Author Disclosures

The authors have reported that they have no relationships relevant to the contents of this paper to disclose.
